# Dietary rice bran promotes resistance to *Salmonella enterica* serovar Typhimurium colonization in mice

**DOI:** 10.1186/1471-2180-12-71

**Published:** 2012-07-04

**Authors:** Ajay Kumar, Angela Henderson, Genevieve M Forster, Andrew W Goodyear, Tiffany L Weir, Jan E Leach, Steven W Dow, Elizabeth P Ryan

**Affiliations:** 1 Department of Clinical Sciences, Colorado State University, Fort Collins, CO 80523, USA; 2 Department of Microbiology, Immunology, and Pathology, Colorado State University, Fort Collins, CO, 80523, USA; 3 Department of Food Science and Human Nutrition, Colorado State University, Fort Collins, CO 80523, USA; 4 Department of Bioagricultural Sciences and Pest Management, Colorado State University, Fort Collins, CO 80523, USA

## Abstract

**Background:**

Dietary rice bran consists of many bioactive components with disease fighting properties; including the capacity to modulate the gut microbiota. Studies point to the important roles of the gut microbiota and the mucosal epithelium in the establishment of protection against enteric pathogens, such as *Salmonella*. The ability of rice bran to reduce the susceptibility of mice to a *Salmonella* infection has not been previously investigated. Therefore, we hypothesized that the incorporation of rice bran into the diet would inhibit the colonization of *Salmonella* in mice through the induction of protective mucosal responses.

**Results:**

Mice were fed diets containing 0%, 10% and 20% rice bran for one week prior to being orally infected with *Salmonella enterica* serovar Typhimurium. We found that mice consuming the 10 and 20% rice bran diets exhibited a reduction in *Salmonella* fecal shedding for up to nine days post-infection as compared to control diet fed animals (p < 0.05). In addition, we observed decreased concentrations of the pro-inflammatory cytokines, TNF-alpha, IFN-gamma, and IL-12 (p < 0.05) as well as increased colonization of native *Lactobacillus* spp. in rice bran fed mice (p < 0.05). Furthermore, in vitro experiments revealed the ability of rice bran extracts to reduce *Salmonella* entry into mouse small intestinal epithelial cells.

**Conclusions:**

Increasing rice bran consumption represents a novel dietary means for reducing susceptibility to enteric infection with *Salmonella* and potentially via induction of native *Lactobacillus* spp.

## Background

*Salmonella* outbreaks are a major health challenge and medical problem around the world. Of the ~2,200 strains, *Salmonella enterica* and *enteridis* cause 75% of total disease incidence [1]. Disease occurrence has resulted in economic burdens of $0.5 to $2.3 billion due to healthcare costs and productivity loss [2]. Emergence of drug resistant *Salmonella* strains is a strong rationale for the development of easily implemented dietary strategies to reduce susceptibility to infection [3,4]. Evidence suggests that presence of some indigestible saccharides and polyphenols in the diet can affect survival and maintenance of gut microflora as well as help prevention of colonization by enteric pathogens [5-7]. For example, non-digestible carbohydrates can be fermented by native gut *Lactobacillus* spp*.* which results in the production of organic acids, such as bacteriocins and hydrogen peroxides. These byproducts are associated with reduced growth of *Salmonella*[8,9]. Therefore, dietary supplementation represents a novel approach to aid in the induction of protective responses against enteric infections.

Little is known regarding the potential impact of whole foods on the colonization of *Salmonella* in the small intestine because traditional biomedical research methods focus on the effect of single nutrients or isolated dietary small molecules [10]. Rice is an important staple food worldwide and the bran portion is typically removed, making rice bran widely available for human and animal consumption. Rice bran contains prebiotic components [11], and is a rich source of bioactive polyphenols, fatty acids and peptides [12-16]. Dietary rice bran intake has been shown to increase the fecal IgA and native gut *Lactobacillus* spp. in mice [17]. Also, rice bran has been found to control gastrointestinal cancers, hyperlipidemia and diabetes in rats [18-21] as well as hypercholesterolemia in humans [22].

The primary goal of this study was to examine the effect of dietary rice bran intake on susceptibility of mice to oral challenge with *Salmonella*. The *Salmonella enterica* serovar Typhimurium strain 14028s was chosen for these studies because it is a translational model of non-lethal, infection in female 129 S6/SvEvTac mice [23]. The protective effect of rice bran against *Salmonella* infection in mice was measured by decreased fecal shedding following oral challenge. These novel findings of rice bran bioactivity have practical implications for developing accessible, affordable and effective dietary public health intervention strategies to reduce *Salmonella* infections worldwide.

## Results

### Effect of dietary rice bran intake on *Salmonella* fecal shedding

**Figure 1:**
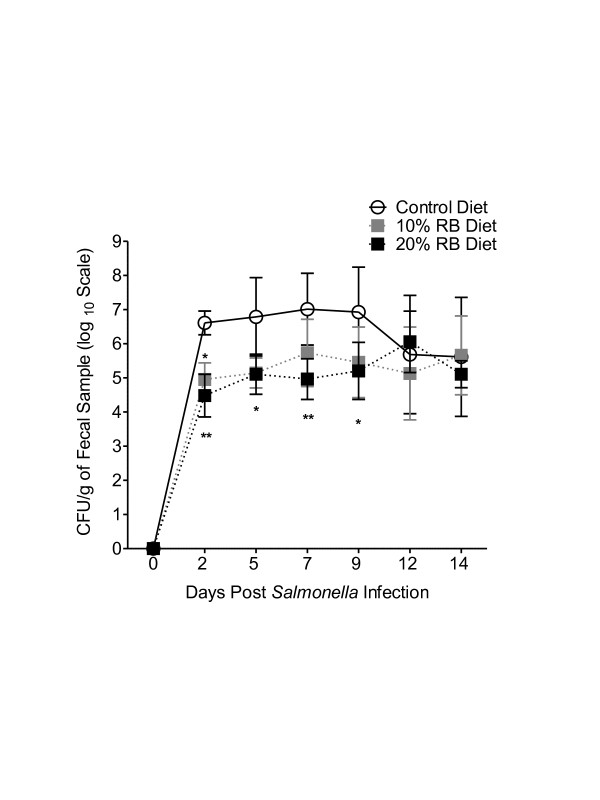
**Effect of dietary rice bran on*Salmonella*fecal shedding of mice.** Fecal shedding was examined in *Salmonella* infected animals fed control, 10% and 20% rice bran diet for 3 weeks (one week prior and 2 weeks post challenge). Data are shown as mean ± standard deviation of mean log_10_ CFU per gram of feces (n = 5 mice/diet group), and data are representative of three independently conducted experiments. Repeated measures ANOVA and post hoc Tukey’s test were applied. Significance is shown by * (*P <* 0.05) and ** (*P <* 0.01).

Daily dietary rice bran supplementation was examined in a mouse model of *Salmonella* infection. Control and rice bran diets were fed to mice for one week prior to oral challenge with *S*. Typhimurium and during infection*.* Mice consuming the rice bran diet showed a time dependent decrease in the fecal shedding of *Salmonella* as compared to control diet animals (Figure 1). More specifically, animals fed the 10% rice bran diet exhibited decreased *Salmonella* fecal shedding by a log_10_ value of 1.66, 1.69 and 1.48 in comparison to animals fed the control diet on days 2, 5 and 9 post infection, respectively (*p <* 0.05). Animals fed the 20% rice bran diet showed a reduction in *Salmonella* fecal shedding by a log_10_ value of 2.13, 1.69, 2.04 and 1.73 in comparison to the animals fed the control diet on days 2, 5, 7 and 9, respectively. No significant difference was observed in *Salmonella* fecal shedding between the 10 and 20% rice bran diet groups. These data demonstrate that pre-feeding dietary rice bran for one week reduced the susceptibility of mice to oral infection with the *Salmonella* pathogen as measured by fecal shedding.

### Effect of dietary rice bran on serum cytokines

**Figure 2:**
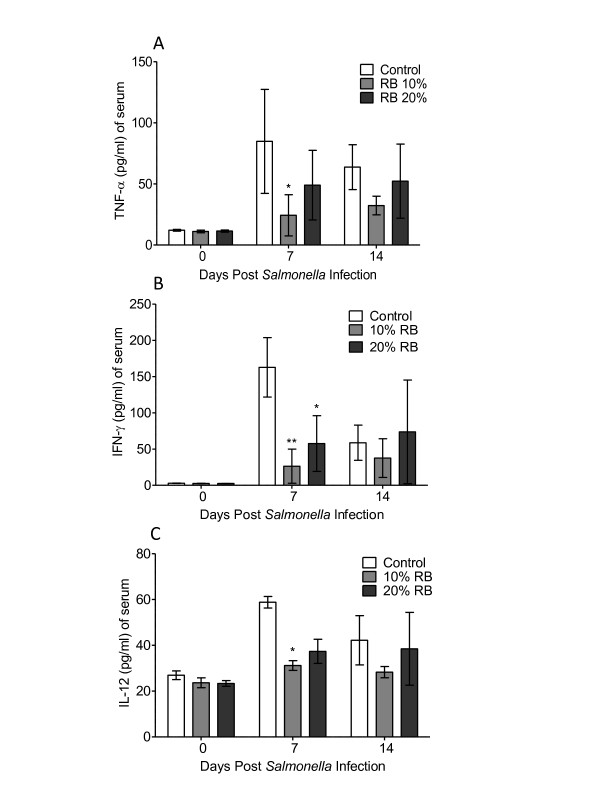
**Effect of dietary rice bran on serum TNF- α, IFN-γ and IL-12 levels in*Salmonella*infected mice.** Blood was drawn at days 0, 7 and 14 following *Salmonella* infection and serum was analyzed for TNF- α (A), IFN-γ (B) and IL-12 (C) levels in control, 10% and 20% rice bran diet groups. Data are shown as mean ± standard deviation of mean (n = 3 mice/diet group). Significance was measured by two-way ANOVA and Bonferroni post hoc test.

Previous research demonstrated that in response to primary *Salmonella* infection, the host immune system releases massive amounts of the cytokines such as TNF-α, IFN-γ and IL-12 locally and systemically [24]. The local inflammatory response has been shown to shift the microbiota composition allowing *Salmonella* the opportunity to efficiently colonize in the gut [25]. Therefore, due to the fact that rice bran mediated a decrease in fecal shedding, we next measured the cytokine level in the serum of mice consuming either the 10 or 20% rice bran diets (Figure 2). Mice fed the 10% rice bran diet for 7 days had decreased serum levels of TNF-α, IFN-γ, and IL-12 by 60.4, 136.3 and 27.6 pg/ml respectively in comparison to animals on the control diet (p < 0.05). Additionally, mice fed the 20% rice bran diet showed decreased levels of serum IFN-γ in comparison to control animals (p < 0.05). These data suggests that rice bran induced suppression of systemic cytokine production may play a role in reducing the colonization of *Salmonella*.

### Effect of dietary rice bran on fecal *Lactobacillus* spp

**Figure 3:**
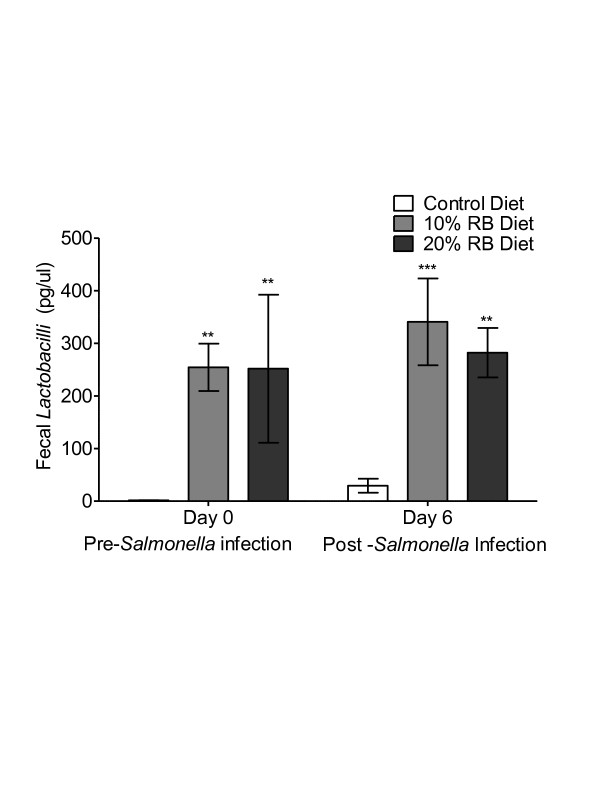
**Effect of dietary rice bran on fecal*Lactobacillus*spp.***Lactobacillus* spp*.* DNA (pg/μl) from fecal pellets of mice before *Salmonella* infection (day 0) and at day 6 (post infection) was determined using qPCR. Error bars indicate standard deviation of mean and * (*P <* 0.05), ** (*P <* 0.01) and *** (*P <* 0.001) denote significant differences in rice bran fed mice from controls (n = 5 mice/diet group). Significance was tested by repeated measures ANOVA and Tukey’s post hoc test.

Members of the genus *Lactobacillus* are potent commensal bacteria with potential for eradication of *Salmonella* infection [26]. Uninfected mice on the 10 and 20% rice bran diets had a 170 and 167-fold higher (p < 0.05) numbers of fecal *Lactobacilli*, respectively, compared to mice on the control diet (Figure 3). Following infection, the levels of fecal *Lactobacilli* remained higher (11- and 9-fold) in the mice consuming the rice bran diets than in the control diet fed mice (Figure 3). These data suggest that rice bran induced changes in gut microbiota may be in part responsible for reduced fecal shedding of *Salmonella*.

### Rice bran extract inhibited *Salmonella* entry and replication in vitro

**Figure 4:**
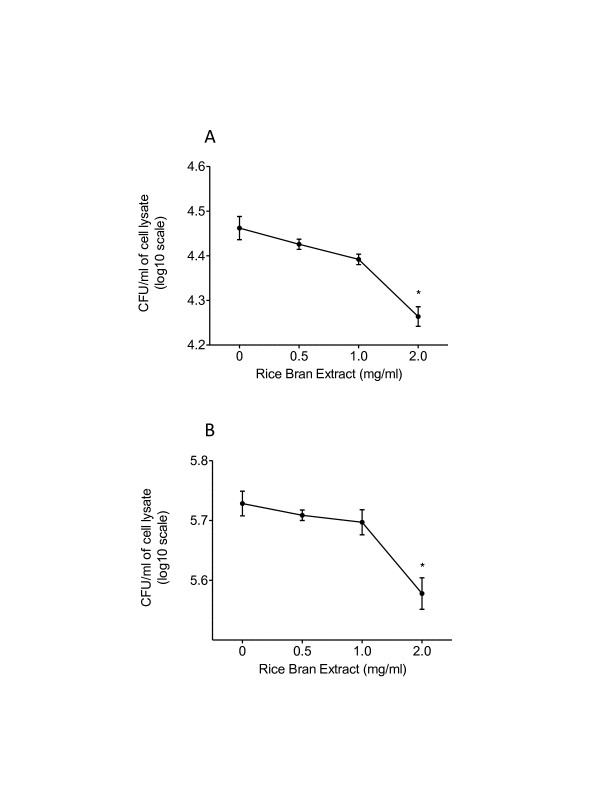
**Effect of rice bran extract on*Salmonella*entry and intracellular replication in MSIE cells.** MSIE cells pre-incubated with rice bran extract (RBE) at doses of 0, 0.5, 1.0 and 2.0 mg/ml for 24 hours, followed by the co-incubation of the RBE with *Salmonella* showed significant inhibition of *Salmonella* entry (A). RBE was tested for effects on intracellular *Salmonella* replication inside MSIE cells for 24 hours (co-incubated with RBE) (B). Bacteria are shown as mean ± standard deviation of mean log_10_ CFU per mL of cell lysate (n = 3). Significance was determined using a nonparametric (Kruskal Wallis) ANOVA, followed by Dunn’s multiple means comparison. Statistical differences denoted by * (*P <* 0.05) and ** (*P <* 0.01).

The ability of *Salmonella* to invade intestinal epithelial cells is an important step involved in the establishment of infection [27]. The ability of rice bran components to interfere with *Salmonella* entry was tested in the mouse small intestinal epithelial (MSIE) cell model. Concentrations of rice bran extract (RBE) that did not affect MSIE cell viability were used (0–2 mg/ml) in these studies (data not shown). RBE (2 mg/ml) reduced the entry of *Salmonella* into MSIE cells by 27% compared to controls (*p <* 0.05) (Figure 4A). The RBE in cell culture media did not kill *Salmonella* directly and therefore did not confound the results of reduced pathogen entry (data not shown).

We next assessed the ability of RBE to inhibit the intracellular replication of *Salmonella* in MSIE cells (Figure 4B). After infection and incubation, extracellular bacteria were removed by washing and antibiotic treatment, and kept for 24 h with RBE. The 2 mg/ml dose of RBE reduced intracellular *Salmonella* replication by 30% (*p <* 0.05) in comparison to control. No direct effect of RBE on *Salmonella* extracellular growth and replication was detected (data not shown). These results suggest that the rice bran extract contains bioactive compounds that block *Salmonella* entry into MSIE cells as well as inhibit intracellular *Salmonella* replication in in vitro model.

We next assessed the ability of RBE to inhibit the intracellular replication of *Salmonella* in MSIE cells (Figure 3B). After infection and incubation, extracellular bacteria were removed by washing and antibiotic treatment, and kept for 24 h with RBE. The 2 mg/ml dose of RBE reduced intracellular *Salmonella* replication by 30% (*p <* 0.05) in comparison to control. No direct effect of RBE on *Salmonella* extracellular growth and replication was detected (data not shown). These results suggest that the rice bran extract contains bioactive compounds that block *Salmonella* entry into MSIE cells as well as inhibit intracellular *Salmonella* replication in in vitro model.

### Rice bran diet components and weight of animals

Dietary rice bran intake did not significantly change the body weight of animals in the experimental and control groups throughout the various studies (data not shown). The total lipid content of the Neptune rice variety is 13.8%; therefore we adjusted the amount of corn oil in the diets to equalize the total fat content in the control, 10% and 20% rice bran diets (Table 1). Also, various dietary components may act as substrates for the gut microflora, and for that reason the total amounts of starch and cellulose were adjusted to balance the macronutrient content across groups.

**Table 1:** Composition of control (AIN93-M) and Rice Bran supplemented mice diets

**Constituents (g/kg)**	**Control**	**10% RB**	**20% RB**
Casein	140	140	140
L-Cystine	1.8	1.8	1.8
Corn Starch	465.7	422.7	377.7
Maltodextrin	155	155	155
Sucrose	100	100	100
Corn Oil	40	19	0
Cellulose	50	29	8
Mineral Mix	35	35	35
Vitamin Mix	10	10	10
Choline Bitartrate	2.5	2.5	2.5
TBHQ*	0.008	0.008	0.008
Rice Bran (RB)	0	100	200

*TBHQ- Tertiary butyl-hydroquinone

## Discussion

In this study, we examined the ability of dietary rice bran to protect mice against an oral challenge with *Salmonella*. Decreased *Salmonella* fecal shedding is a reliable marker for reduced susceptibility to infection [28-30] and was used herein to determine whether dietary rice bran supplementation reduced susceptibility to *Salmonella* infection. Fecal shedding of *Salmonella* from orally challenged mice fed 10 and 20% rice bran diets was significantly reduced as compared to control diet (Figure 1). Consistent with previous research, the highest number of fecal *Salmonella* in the control diet fed mice was observed on day 7, followed by a reduction in *Salmonella* numbers on days 8–13 (Figure 1) [28]. *Salmonella* fecal shedding in rice bran fed mice was consistently lower than control diet fed mice until day 9-post infection.

We chose this mouse model of *Salmonella* infection over other models because the 129 S6/SvEvTac mice do not die from disseminated *Salmonella* infection due to presence of both functional copies of the *nramp1* gene whereas other strains would die within 7–14 days of inoculation [28]. Although our data suggested that rice bran supplementation decreased *Salmonella* invasion in the ileum, Peyer’s patches and mesenteric lymph node of the rice bran fed mice, these values were not significant ( Additional file 1: Figure S1). Thus, the rice bran diet reduced *Salmonella* fecal shedding may be a result of the induction of increased colonization resistance in the intestinal lumen as opposed to the increased horizontal transfer of *Salmonella* into the tissues [31].

Gut inflammation resulting from *Salmonella* presence favors the colonization and growth of the *Salmonella* because of changes in gut ecology and environment [25]. Local inflammation in the intestine occurs in conjunction with a massive systemic release of TNF-α, IFN-γ and IL-12 [24,32,33]. The rice bran fed mice showed a significant reduction in serum inflammatory cytokines associated with *Salmonella* infection, namely TNF-α, IFN-γ and IL-12 (Figure 2A-C). The presence of *Salmonella* antigens in the lumen is in part responsible for inducing the inflammatory cytokines in control diet fed animals. Therefore, a reduced *Salmonella* antigen load in the lumen of rice bran fed mice may have diminished this inflammatory response. Determining the mucosal immune cells involved in the development of local and systemic inflammation by *Salmonella* in these mice will be important for understanding the mechanisms by which rice bran modulates the inflammatory response.

Given that *Salmonella* induces changes in the gut microbiome [25,34], we next explored differences in the gut microbial communities between control and rice bran fed mice as a plausible mechanism for the reduced colonization of *Salmonella* (Figure 1). Our exploratory data showed increased *Firmicutes* in rice bran diet fed animals as compared to control animals before infection (Data not shown). The phylum *Firmicutes* contains the genus *Lactobacillus* and rice bran fed animals demonstrated a ~170 fold increase in fecal *Lactobacillus* spp. content as compared to control before infection (Figure 3). Probiotic *Lactobacillus* spp. protect against *Salmonella* infection through production of lactic acid that modulates bacterial virulence gene expression and can help maintain tight junctions of mucosal epithelial cells [35-37]. Changes in the gut microbiota by dietary rice bran warrant a separate study to explore this novel mechanism for prevention and reduced susceptibility to *Salmonella* infection.

**Figure 5:**
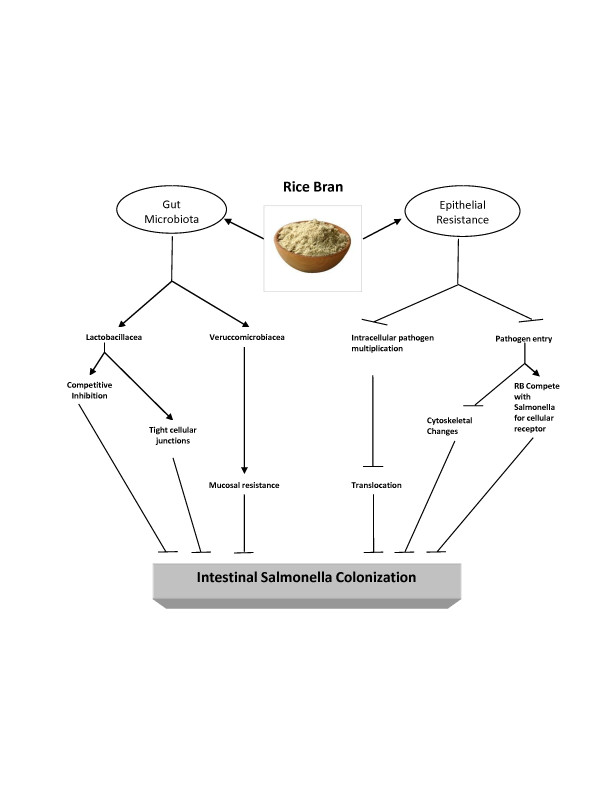
**Potential mechanisms involved in dietary rice bran induced reduction in susceptibility to*Salmonella*infection.** Rice bran may inhibit *Salmonella* colonization via modulation of gut microbiota, preventing cellular entry of *Salmonella*, and inhibiting intracellular replication.

Rice bran is a collection of numerous bioactive components [17] that may exhibit multiple mechanisms of action for protection against enteric pathogens. Methanol extracts contain bioactive polyphenols and fatty acids from rice bran [38], and were used for the treatment of MSIE cells in vitro. RBE reduced the cellular entry of *Salmonella* by 27% in comparison to control (Figure 4A). In addition to reduced *Salmonella* entry, RBE also decreased intracellular *Salmonella* replication by 30% (Figure 4B). These in vitro findings merit further investigation of the rice bran effects on the epithelium in vivo. Rice bran phytochemicals may inhibit pathogen entry and intracellular replication of *Salmonella* either by modulating the epithelial cytoskeleton, blocking receptors, altering the cellular microenvironment, and/or by influencing virulence gene expression [39,40]. Additional mechanisms may include increased production of bile and gastric acids and increased intestinal motility by dietary rice bran. Future studies are warranted to elucidate these mechanisms and to determine the specific combinations of bioactive rice bran components responsible for protection against infection (Figure 5). Our findings provide a rationale for biomedical scientists to work closely with rice crop scientists for advancing our understanding of rice bran-microbe interactions. These findings set the stage for additional work with the rice industry, public health and veterinary nutritionists to determine whether the dietary supplementation of rice bran offers greater mucosal protection against enteric infections in people and animals.

## Conclusions

Our study has indicated a potential use for dietary rice bran to mitigate *Salmonella* infection. Increasing consumption of rice bran represents a promising and novel means for reducing susceptibility to enteric infection with *Salmonella*, potentially through the modulation of native gut *Lactobacillus* spp. Further investigation in animal models and human clinical studies will be necessary to elucidate mechanisms of action and physiological importance of dietary rice bran supplementation against enteric infections.

## Methods

### Animals and feeding schedule

Four-to-six weeks-old female 129 S6/SvEvTac (Taconic Farms, Germantown, NY) mice were randomly divided into 3 groups (n = 5 in each group) and housed with a 12-hour light/dark cycle at 20–25°C. Animals were provided water and fed a maintenance diet AIN-93 M (Harlan Teklad, Madison, WI) *ad libido* for three weeks. After 3 weeks, mice were randomized into Group 1- AIN-93 M control diet, Group 2–10% rice bran diet, or Group 3–20% rice bran diet. The Animal Care and Use Committee at Colorado State University approved all mouse protocols (Protocol number 09-1457A).

### Bacterial infection

*Salmonella enterica* serovar Typhimurium strain 14028s was a generous gift from Dr. Andres Vazquez-Torres (University of Colorado). *Salmonella* was grown in LB broth (Sigma Aldrich) at 37°C overnight to obtain stationary phase cultures, 15% glycerol (Fisher Scientific) was added and stocks were stored at −80°C. Frozen *Salmonella* stock was thawed and diluted with PBS to a final concentration of 2 × 10^7^ CFU/ml. Mice were infected with ~2 × 10^7^ CFU in a total volume of 200 μl using a 25-gauge gavage needle. Each inoculum used for oral infection was plated on MacConkey agar (BD Biosciences) to confirm bacterial concentration.

### Diet composition

Rice bran used in these studies was provided as a gift from Dr. Anna McClung at USDA-ARS Dale Bumpers National Rice Research Center (Stuttgart, AK). Diets were formulated to match macronutrients (e.g. protein, carbohydrates) across groups. Differences in macronutrient composition were balanced using purified diet components. The percent of rice bran incorporated into the diet is expressed as g/100 g of diet. Harlan mixed and made pellets of rice bran containing diets using AIN-93 M purified components. The composition of rice bran containing diets was calculated based on published reports [41-43] that demonstrated chronic disease fighting activity. Diet formulations are shown in Table 1. The Neptune rice variety was chosen for its availability.

### Fecal collection and processing

Fecal pellets were collected and body weights were recorded on day 0 before oral challenge, and on days 2, 5, 7, 9, 12 and 14-post infection. Mice were kept in Tupperware for 30 minutes and pellets from each mouse were weighed and diluted with PBS. After homogenization, fecal matter was serially diluted and plated on MacConkey agar (BD Biosciences) with 50 μg/ml of kanamycin (Fisher Scientific). Agar plates were incubated at 37°C under humid conditions for 24 hours and bacteria were counted as CFU/g of fecal matter. Feces from rice bran fed, uninfected mice were plated on MacConky agar with kanamycin and no *Salmonella* CFU was detected in the plates. Morphology of *Salmonella* colony in pure culture and infected feces were similar.

### Blood and tissue collection

Blood was collected by tail vein (before infection) or cardiac puncture (before necropsy) using 4% Isoflurane (Attane Isoflurane USP, Minard Inc) in anesthesia machine with oxygen at a flow rate of 0.1 L/min. Serum separator tubes (BD Microtainer) were centrifuged at 7500 g for 10 minutes and stored at −20°C. Spleen, liver, ileum (distal 10 cm), mesenteric lymph nodes and Peyer’s patches were harvested, thoroughly washed with PBS, weighed and transferred to bags (Whirl-Pack, Nasco) and homogenized in stomacher (Seward Stomacher 80, Biomaster Lab Systems). Serial dilutions of homogenized tissues were plated on MacConkey agar with 50 μg/ml of kanamycin.

### Serum cytokine analysis

Serum cytokines (TNF-α, IFN-γ and IL-12) were analyzed by cytometric bead array assay using the mouse inflammation kit (BD Biosciences) and the assay was performed according to the manufacturer’s instructions. Flow cytometry was performed using a Cyan ADP flow cytometer and Summit software (Beckman Coulter), and FlowJo software (TreeStar Inc) was used for analysis and quantification of serum cytokine data.

### Cell culture conditions

Mouse small intestine epithelial cells (MSIE) were a generous gift from Dr. Robert Whitehead at Vanderbilt University and the Ludwig Institute for Cancer Research [44]. Briefly, MSIE cells were grown using published methods in RPMI 1640 media supplemented with 2.05 mM L-Glutamine (Hyclone Laboratories). RPMI media was also supplemented with heat inactivated 10% FBS (Atlas Biologicals), 1% antibiotic (Penicillin and Streptomycin) and antimycotic (Amphotericin) solution (Cellgro, Mediatech Inc), 0.1% Thioglycerol Hydrocortisone (Sigma), 0.004% IFN-γ (Peprotech USA), 0.023% Insulin (Regular Human Insulin, Novo Nordik). Cells were grown in 75 cm^2^ flasks and trypsinized at 80% confluence. Cells were seeded overnight in a 6 well plate at a density of 2 × 10^5^ cell/well. After 12 hours media was aspirated and fresh media was added with rice bran extracts for 24 hours at 37°C and 5% CO_2_ and 95% humidity.

### Rice bran extraction

Crude rice bran cannot be reliably tested in cellular assays, and was therefore extracted with 80% methanol to obtain a mixture of rice bran phytochemicals and called a rice bran extract (RBE). Briefly, rice bran (Neptune variety) was removed from the grain and heat stabilized at 110°C for 3 minutes. Ice-cold, 80% methanol was added, vortexed and incubated at −80°C for one hour. Following centrifugation at 1500 g for 5 minutes, the supernatant was removed. Methanol was dried by vacuum centrifugation (SpeedVac Concentrator, Thermo Savant Model RT-100). Dried rice bran extract was weighed and then re-suspended with cell culture media to the appropriate doses for treatment of MSIE cells.

### *Salmonella* entry and replication

*Salmonella* entry assay was done according to previously published protocol [45]. This assay measures the total number of *Salmonella* (the bacteria that is surface attached plus the *Salmonella* internalized in the cell). MSIE cells were grown and treated with RBE for 24 hours. Media was aspirated and cells were re-incubated with fresh media containing *Salmonella* and RBE. Frozen stock of *Salmonella* was mixed in RPMI media at a Multiplicity of Infection (MOI) of 100–120 in the presence (co-incubation with *Salmonella*) or absence of RBE. After 30 minutes of incubation, media was aspirated, and MSIE cell monolayer was washed with PBS twice to remove extracellular bacteria. Fresh media was added to cells for additional 1 hour. There were 2 additional cycles of washing with fresh media plus 50 μg/ml of gentamicin (Sigma-Aldrich) following 1-hour incubations under the same conditions with 5 μg/ml of gentamicin. Media was aspirated and cell monolayer was washed with PBS twice to remove extracellular gentamicin. The cell monolayer was placed in 1 ml of buffer (PBS containing 1% TritonX-100 and 0.1% SDS) for 5 minutes. The contents were mixed by pipetting and serially diluted on MacConkey agar plates (BD Biosciences) with 50 μg/ml of kanamycin (Fisher Scientific) and incubated at 37°C for 24 hours. Colonies were counted and presented per ml of cell lysate. Intracellular *Salmonella* replication was measured in cells incubated with 5 μg/ml of gentamycin and RBE for 24 hours. Cells were lysed and plated on agar media to enumerate the total CFU count [46].

### Cell viability

Cell viability was determined using alamarBlue (Invitrogen). Briefly, cells were seeded in a 96 well plate at 2x10^5^/ml. After 6 hours of cell adherence, cells were treated in the presence and absence of RBE for 24 hours at 37°C, 5% CO_2_ in maintenance media. Supernatant was removed and alamarBlue was added to media (20 μg/ml). Fluorescence was detected at excitation: 530/25; emission: 590/35 in ELISA plate reader (Bio-Tek Synergy HT, Winooski, VT).

### Bacterial quantitation

RBE doses of 0, 1, 2, 5 and 10 mg/ml were tested for direct effects on *Salmonella* viability. Bacteria was added to media at a concentration of 2 × 10^7^ CFU/ml and incubated for 6 hours at 37°C. Bacterial suspension was serially diluted, plated on agar plates and counted after 24 hours incubation.

### Quantitative PCR for *Lactobacillus* spp

DNA was extracted from fecal pellets of control and rice bran fed mice before and after *Salmonella* challenge using a MoBio Powersoil DNA extraction kit (MoBio, Carlsbad, CA). A dilution of DNA from pure cultures of *Lactobacillus rhamnosus* was used to generate standard curves and DNA from *Pseudomonas aeruginosa* were run as a negative control to ensure primer specificity. DNA was quantified by Nanodrop (Thermo Fisher Scientific) and diluted to 5 ng/μl. Real time PCR primers were used from Malinen et al. [47] for amplification of *Lactobacillus* spp. Samples were run on an ABI Prism 310 thermocycler (Applied Biosystems) using the following program: 95°C for 3 min 30 s followed by 30 cycles of 95°C for 15 s, 58°C for 20 s 72°C for 30 s and melt curves were generated by 95°C for 1 min followed by eighty 10 s repeats at set point temperatures incrementally decreasing by 0.5°C.

### Statistical analysis

Data was analyzed using Graphpad Prism5 Software. Experiments were repeated a minimum of three times. Raw data were log transformed into a log_10_ scale for CFU analysis and repeated measures ANOVA and post hoc Tukey’s test were used for *Salmonella* fecal shedding and fecal *Lactobacilli* measures. Inflammatory cytokines were analyzed using two -way ANOVA and Bonferroni post hoc test. A nonparametric ANOVA (Kruskal Wallis) was performed, followed by Dunn’s test for in vitro *Salmonella* assays. Significance was determined for all studies at *P* <0.05.

## Competing interests

The authors disclose no conflicts of interest.

## Authors’ contributions

The experiments were conceived and designed by AK, SD and ER. AK, AH, AG, TW and GF performed the experiments. AK, TW, JL, SD and ER analyzed data. JL, TW, SD and ER contributed reagents, materials and analysis tools. AK, SD, AH and ER wrote the paper. All authors read and approved the final manuscript.

## Supplementary Material

Additional file 1 **Figure S1: Effect of dietary rice bran on*Salmonella*tissue invasion.***Salmonella* infected animals were sacrificed on days 7 (Figure S1A-C) and 14 (S1 D-F) following oral challenge and selected tissues were homogenized and plated for enumeration of bacteria. Trends in the data indicates that rice bran supplementation decrease *Salmonella* translocation into the ileum, Peyer’s patches and mesenteric lymph nodes but failed to achieve statistical significance. Click here for file
