# Supplementary figures and images for: Dietary rice bran promotes resistance to Salmonella enterica serovar Typhimurium colonization in mice
Source: BMC Microbiol. 2012 Jul 4;12:71. doi: 10.1186/1471-2180-12-71 (PMC3390288; doi:10.1186/1471-2180-12-71)

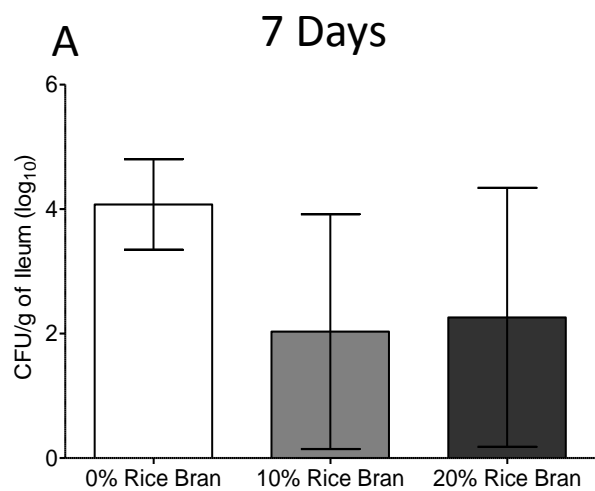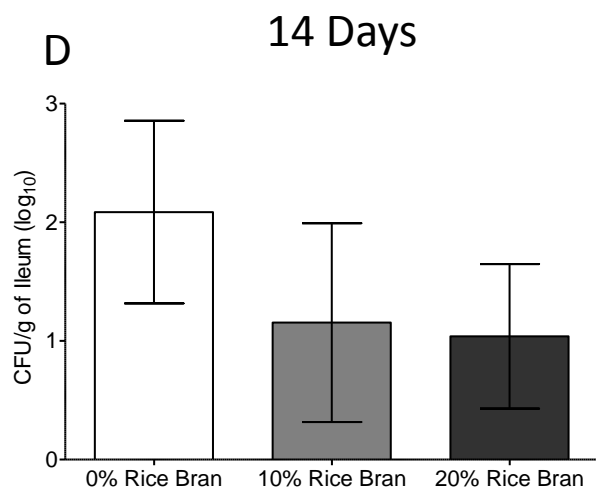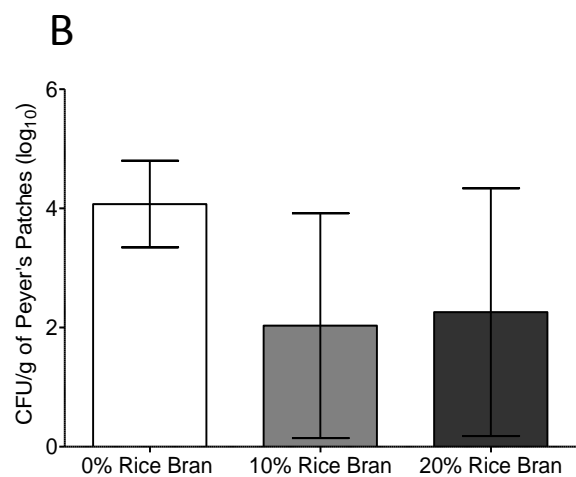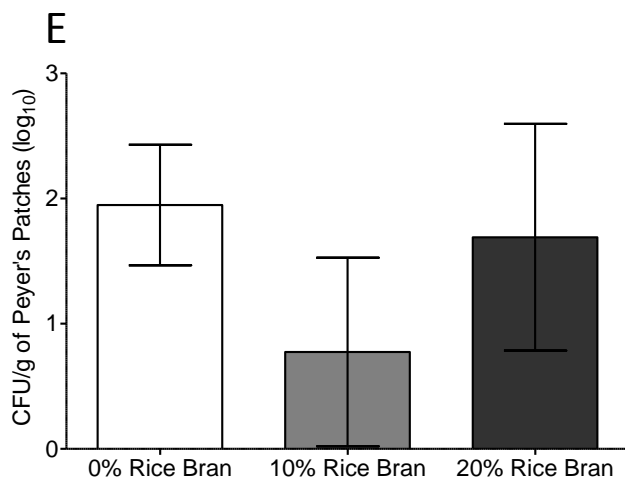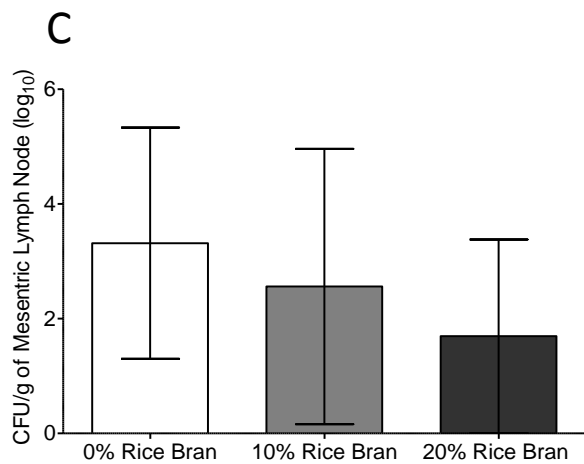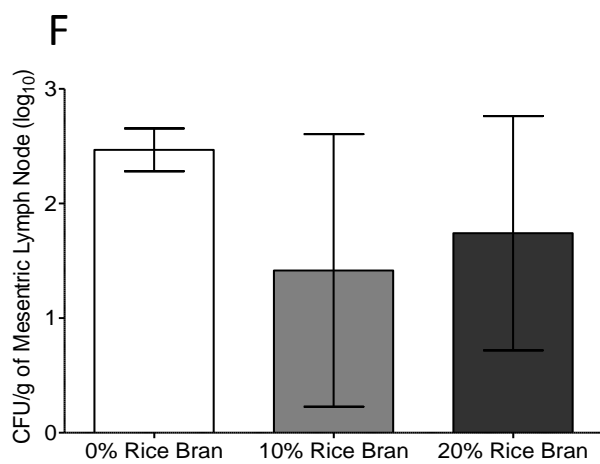

Supplement: Additional file 1 — Figure S1: Effect of dietary rice bran onSalmonellatissue invasion.Salmonella infected animals were sacrificed on days 7 (Figure S1A-C) and 14 (S1 D-F) following oral challenge and selected tissues were homogenized and plated for enumeration of bacteria. Trends in the data indicates that rice bran supplementation decrease Salmonella translocation into the ileum, Peyer’s patches and mesenteric lymph nodes but failed to achieve statistical significance. [file 1471-2180-12-71-S1.pdf]
